# Dual-Frequency Ultrasound-Assisted Glycation Modulates Beef Myofibrillar Protein Gels and Inhibits Lysinoalanine Formation

**DOI:** 10.3390/gels12080669

**Published:** 2026-07-25

**Authors:** Zhaoli Zhang, Haochen Hu, Silu Chen, Zhikun Yang, Ronghai He, Yang Wang, Xiangren Meng

**Affiliations:** 1 Key Laboratory of Chinese Cuisine Intangible Cultural Heritage Technology Inheritance, College of Tourism and Culinary Science, Yangzhou University, Yangzhou 225127, China; zhaoli@yzu.edu.cn (Z.Z.); huhczm_0305@163.com (H.H.); yangzhikun@yzu.edu.cn (Z.Y.); 2 College of Food Science and Engineering, Yangzhou University, Yangzhou 225127, China; mz120242237@stu.yzu.edu.cn; 3 School of Food and Biological Engineering, Jiangsu University, Zhenjiang 212013, China; heronghai@163.com

**Keywords:** dual-frequency ultrasound, glycation, Maillard reaction, lysinoalanine inhibition, rheological properties, emulsion stability, protein oxidation

## Abstract

Ultrasound-assisted xylose glycation is found to be an effective method to improve the gel properties, functional properties and structural characteristics of meat proteins, and to some extent minimize the generation of protein cross-linking. The effects of dual-frequency ultrasound-assisted (20/25, 20/28, 25/28, 20/40, 25/40, 28/40, 28/68, and 40/68 kHz) xylose glycation on beef myofibrillar proteins (MPs) were evaluated in the study. Compared with the control, dual-frequency ultrasound promoted glycation in a frequency-dependent manner. The highest grafting degree was observed at 20/25 and 25/40 kHz, reaching 37.05% and 38.47%, respectively. For gel-type applications, 25/28 kHz ultrasound provided the most balanced performance. It reduced cooking loss from 79.80% to 73.77%, increased hardness from 1388.18 to 1743.38 g, and increased chewiness from 673.03 to 939.84 g. This treatment also showed favorable emulsifying behavior. The strongest LAL inhibition occurred at 25/40 kHz, where LAL decreased from 7961.24 to 4868.88 mg/kg, corresponding to a 38.8% reduction. Structural analyses indicated that ultrasound-assisted glycation induced MP unfolding, thiol exposure, hydrophobic rearrangement, and oxidative modification to different extents. Overall, 25/28 kHz may be more suitable when balanced gel quality, emulsifying performance, and safety control are required. In contrast, 25/40 kHz may be preferred when LAL reduction is the primary objective.

## 1. Introduction

Beef is valued for its high protein and low fat contents, making it a preferred choice among several consumers. Myofibrillar proteins (MPs) constitute the primary structural proteins in beef and play a key role in determining the structure, emulsifying capacity, and gelation characteristics of meat products [1]. However, conventional processing methods, involving heat, alkaline conditions, and mechanical stress, can readily induce conformational destruction of MPs, resulting in reduced functionality, particularly solubility [2]. Furthermore, the gelation behavior of MPs is closely linked to the water-holding capacity, texture, and overall quality of meat products [3]. Consequently, the development of mild and efficient protein modification techniques to improve the structure, gelation behavior, and functional properties of MPs remains a crucial research priority in meat product science.

Glycation is defined as the non-enzymatic covalent binding of reducing sugars to proteins, a process that can substantially improve the solubility, emulsifying properties, and thermal stability of proteins [4]. During glycation, proteins and carbohydrates can be conjugated using either dry- or wet-heating methods. Among different reducing sugars, xylose has been frequently used in protein glycation systems because its small molecular size and relatively high Maillard reactivity facilitate the interaction between carbonyl groups and reactive amino groups in proteins. Previous studies have also shown that xylose-mediated glycation can modify protein conformation and improve functional properties [5]. Ma et al. [6] reported that under dry or wet conditions, protein and carbohydrate molecules underwent grafting by condensation, involving the α-amino and ε-amino groups of proteins, thereby enhancing their functional characteristics. However, the traditional wet-heating methods tend to induce protein denaturation and aggregation, while dry-heating methods are limited by low reaction efficiency [4]. Recently, physical field-assisted technologies, including microwave treatment [7], high-pressure reactors [8], and ultrasound [9], have provided novel approaches to facilitate glycation reactions. Among these technologies, ultrasonication is notable for its cavitation, mechanical shear, and thermal effects. These effects enhance mass transfer, promote protein–carbohydrate interactions, alter protein conformation, and accelerate glycation [10,11].

Previous studies have demonstrated that single-frequency ultrasound can effectively promote protein glycation and improve its functional characteristics [12]. Compared with single-frequency ultrasound, dual-frequency ultrasound can generate more complex cavitation, acoustic streaming, and mechanical shear effects, which may enhance mass transfer, promote protein unfolding, and improve the accessibility of reactive groups during glycation. Therefore, dual-frequency ultrasound was selected in this study as a potential strategy to coordinate protein structural regulation, functional improvement, and LAL inhibition. However, the use of multi-frequency ultrasound in treating complex meat proteins such as MPs, in this regard, is yet to be systematically investigated. Under alkaline conditions, proteins tend to form lysinoalanine (LAL), a non-natural amino acid produced by the cross-linking of dehydroalanine with lysine residues. This process reduces protein digestibility and poses potential toxicological risks [13]. Earlier studies have demonstrated that ultrasonication could be used to alter protein conformation and the distribution of carbohydrate molecules, and consequently control or inhibit the formation of LAL in the macromolecule [14,15]. Nevertheless, most of these studies have explored the influence of ultrasonication on protein functionality, structural characteristics, and LAL formation independently, while limited attention has been given to gelation behavior under the same modification system. Different frequency combinations may exhibit distinct or even mutually restrictive effects on protein structure, gel properties, emulsifying performance, and LAL inhibition. Accordingly, achieving a balance between gelation behavior, functional improvement, structural regulation, and the inhibition of LAL formation remains a critical yet unresolved scientific issue.

The present study examined the effects of dual-frequency ultrasound-assisted glycation on beef MPs at eight frequency combinations (20/25, 20/28, 25/28, 20/40, 25/40, 28/40, 28/68, and 40/68 kHz). Gelation behavior, functional properties, structural characteristics, emulsifying performance, and LAL formation were evaluated. It was hypothesized that dual-frequency ultrasound-assisted glycation would regulate protein unfolding, protein–xylose conjugation, and oxidative modification in a frequency-dependent manner, thereby modulating MP functionality and LAL formation. By integrating gelation behavior, functional modification, structural regulation, and LAL inhibition within the same alkaline MP–xylose glycation system, this study aimed to identify frequency conditions that balance quality improvement and safety control. The results provide a theoretical basis for advancing meat protein modification technologies aimed at enhancing product quality and ensuring safety.

## 2. Results and Discussion

### 2.1. Grafting Degree (DG)

**Figure 1:**
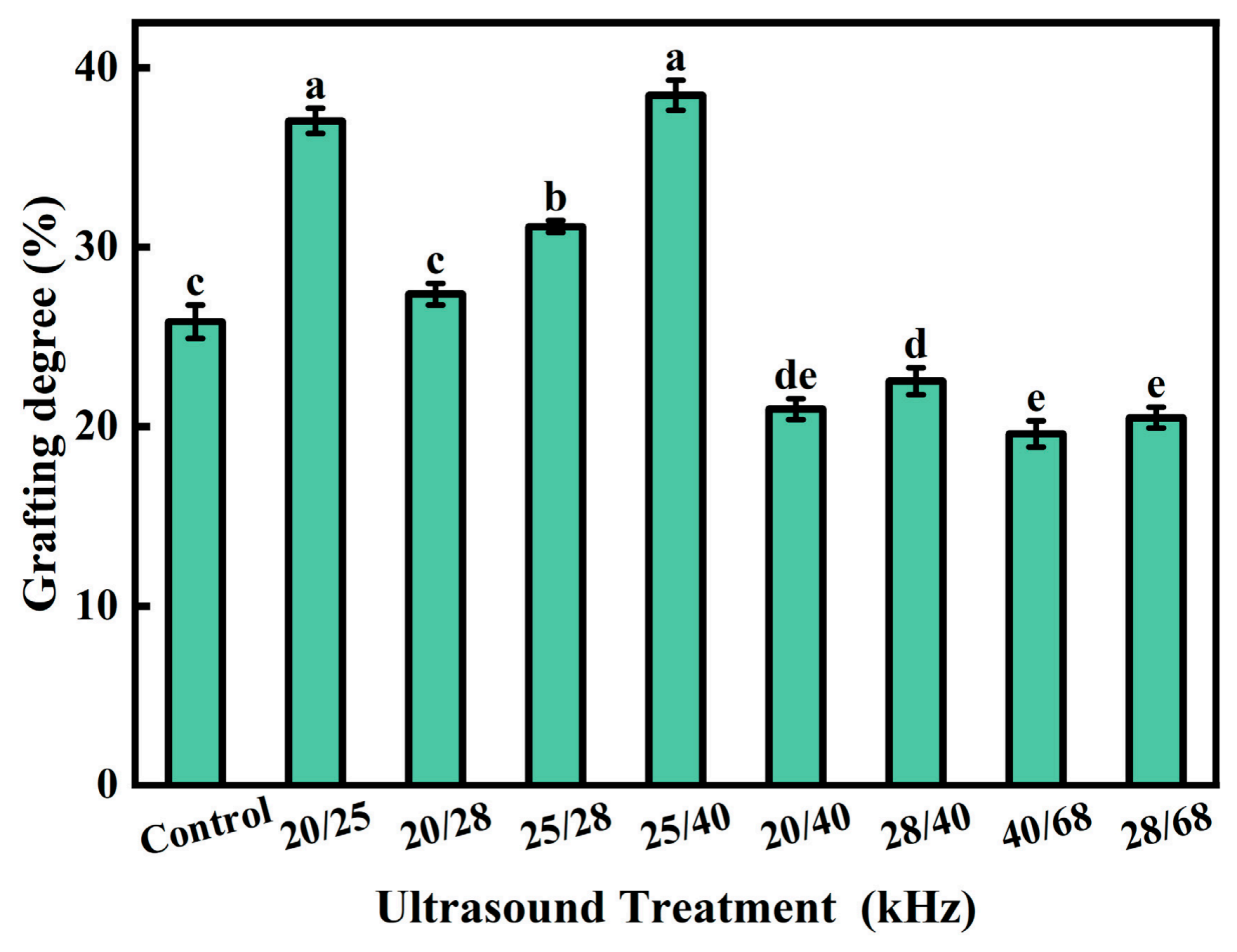
Effects of dual-frequency ultrasound-assisted glycation on the degree of grafting in beef MPs. Different lowercase letters indicate significant differences (*p* < 0.05).

The DG is commonly used to evaluate the progress of the Maillard reaction [16]. As shown in Figure 1, different dual-frequency ultrasound combinations exhibited a strong impact on the DG of the beef protein–xylose system. The DG values under low-frequency combinations were markedly higher than those under high-frequency treatments. The 20/25 and 25/40 kHz treatments showed the strongest grafting-promoting effects. This might be attributed to the stronger cavitation and mechanical shear effects generated by low-frequency ultrasound, which promoted the unfolding of beef MPs and exposed more reactive amino groups for glycation. In contrast, high-frequency combinations may have induced protein re-aggregation or steric hindrance, thereby reducing the accessibility of amino groups and limiting the grafting reaction [17]. Overall, ultrasound-assisted treatment accelerated the Maillard reaction by enhancing energy transfer through thermal and cavitation effects. It also promoted protein unfolding and increased the availability of reactive sites [18]. It should also be noted that protein oxidation may occur concurrently with glycation under some low-frequency ultrasound treatments. Therefore, the increased DG may reflect enhanced protein–xylose conjugation together with changes in amino group availability caused by oxidative modification.

### 2.2. Gel Properties of Beef MPs

#### 2.2.1. Gel Color

The results showed that dual-frequency ultrasound-assisted glycation had no significant effect on the color properties of beef myofibrillar protein gels (Table 1). Specifically, no notable variations were observed in the L* and b* values of the treatment groups, suggesting that glycation combined with different ultrasonication frequency modes did not markedly affect the lightness or yellowness of the protein–xylose gel system. A significant decrease in the a* value was recorded only in the 28/68 kHz group, which might be attributed to intensified cavitation and mechanical effects under specific frequency combinations. This may have caused localized protein oxidation, thereby reducing gel redness and weakening color stability. In contrast, the 25/28 kHz group exhibited more stable L*, a*, and b* values, with only minor fluctuations in each parameter. To further evaluate overall color variation, Δ*E* values relative to the control were calculated and added to Table 1. The Δ*E* values ranged from 0.65 to 2.17, with the lowest value observed in the 25/28 kHz group and the highest value observed in the 28/40 kHz group. These results indicated that the overall color deviation caused by dual-frequency ultrasound-assisted glycation was relatively small. This suggested that 25/28 kHz was more favorable for maintaining overall color coordination. This outcome is consistent with the findings reported by Wang et al. [19].

#### 2.2.2. Cooking Loss

Cooking loss reflects the water-holding capacity of the gel system during thermal processing. Dual-frequency ultrasound-assisted glycation significantly affected the cooking loss of beef MP–xylose gels (Table 1). Among the frequency modes, the 25/28 kHz group exhibited the lowest cooking loss, indicating that the frequency combination was the most effective in improving the water-holding capacity of the gel upon heating. The 28/40 kHz group also showed relatively lower cooking loss, whereas the 20/28 kHz and 25/40 kHz groups presented higher cooking losses. These results suggested that not all dual-frequency combinations could effectively be used to enhance gel quality. Only appropriate dual-frequency ultrasound combinations can promote the moderate unfolding of protein molecules and their subsequent ordered intermolecular aggregation, thereby facilitating the formation of a denser and more homogeneous three-dimensional network structure. This structural enhancement increased the binding capacity of the system for free and immobilized water, ultimately minimizing juice loss during the heating process [20].

#### 2.2.3. Texture Properties

Dual-frequency ultrasound-assisted glycation significantly influenced the textural profile of beef MP–xylose gels (Table 1). Overall, treatments at 20/25, 25/28, 28/40, 28/68, and 40/68 kHz increased gel hardness, with the 40/68 kHz group exhibiting the highest value. The cohesiveness of the 20/25 kHz group increased significantly, indicating a substantial improvement in internal gel stability. However, the 20/28 kHz group showed relatively minor changes across all parameters, suggesting that the improvement in texture was not pronounced. The 20/40 kHz treatment did not enhance hardness and showed a decreasing trend in springiness. Although the 25/40 kHz group demonstrated relatively high springiness, the increases in hardness, and chewiness were not significant. The 25/28 and 40/68 kHz groups revealed not only an increase in hardness but also significant improvements in chewiness. This observation may be attributed to the formation of a dense gel network with strong resistance to deformation [21]. Among the various treatments, the 25/28 kHz treatment exhibited the most balanced gel quality profile. While the 40/68 kHz group demonstrated the highest hardness value, the hardness of the 25/28 kHz group was statistically comparable and was associated with the lowest cooking loss and increased chewiness. This indicates a more advantageous equilibrium between gel strength and water retention. This finding is generally consistent with previous studies suggesting that appropriate combinations of ultrasound frequencies contribute to the improvement of protein gel textural properties [19].

#### 2.2.4. Dynamic Rheological Properties

**Figure 2:**
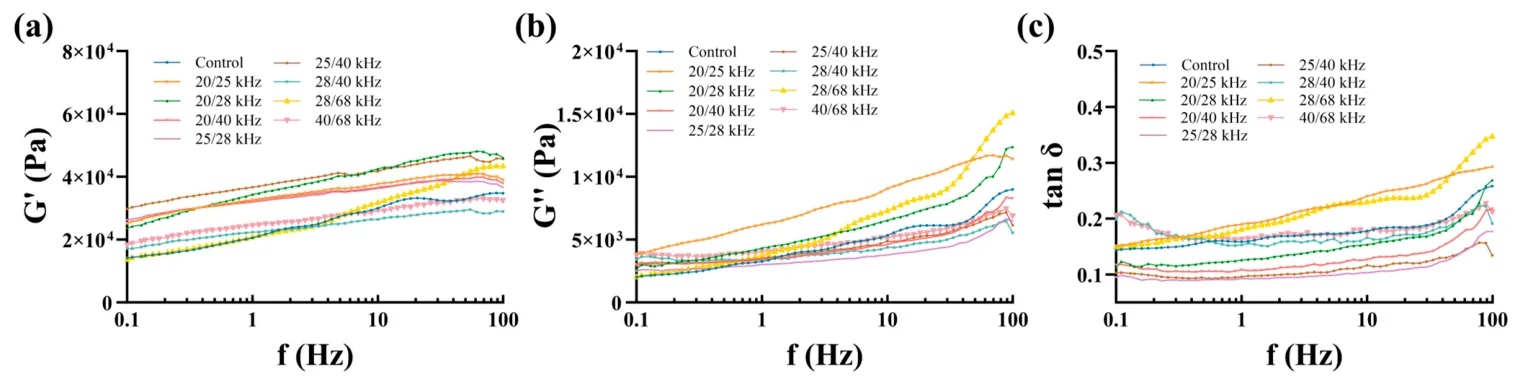
Dynamic rheological properties of formed beef MP–xylose gels after dual-frequency ultrasound-assisted glycation: (**a**) storage modulus (G′), (**b**) loss modulus (G″), and (**c**) loss tangent (tan δ).

Figure 2 shows that dual-frequency ultrasound-assisted glycation significantly affected the rheological properties of beef MP–xylose gels. Across the entire frequency range, G′ values were considerably higher than G″ for all treatment groups and increased with oscillation frequency, implying the formation of a three-dimensional gel network dominated by elastic behavior. Compared with the control, all ultrasound-treated samples exhibited varying degrees of increase in G′ and G″ at low frequencies. The results indicated that dual-frequency ultrasound enhanced the viscoelasticity of the gel system. This observation is consistent with the report by Chen et al. [22].

The 20/25 kHz group exhibited relatively high G′ and G″ values in the low- to intermediate-frequency range, while tan δ decreased markedly with increasing frequency. The 25/28 and 25/40 kHz treatments showed overall lower tan δ values, suggesting that elasticity dominated these systems. For the 25/28 kHz group, the lower tan δ was accompanied by higher hardness and chewiness, supporting the formation of a more stable and denser network structure. In contrast, the 25/40 kHz group showed relatively high modulus and low tan δ, but only limited improvement in textural parameters, indicating that a frequency-dependent decrease in tan δ may also reflect a more frequency-sensitive or less uniform gel structure. Overall, the rheological and textural profile data, although not entirely consistent, were generally correlated. This implies that the macroscopic texture of the gel may also be influenced by network uniformity and internal structural characteristics [23].

It should be noted that the control sample was subjected to the same xylose addition, pH 12.5 shift, heating temperature, and reaction time as the ultrasound-treated samples, but without ultrasonication. Therefore, the differences in gel properties between the control and ultrasound-treated groups reflect the additional regulatory effect of dual-frequency ultrasound on the alkaline glycation system, rather than the effect of pH shift alone.

### 2.3. Functional Properties

#### 2.3.1. Solubility

**Figure 3:**
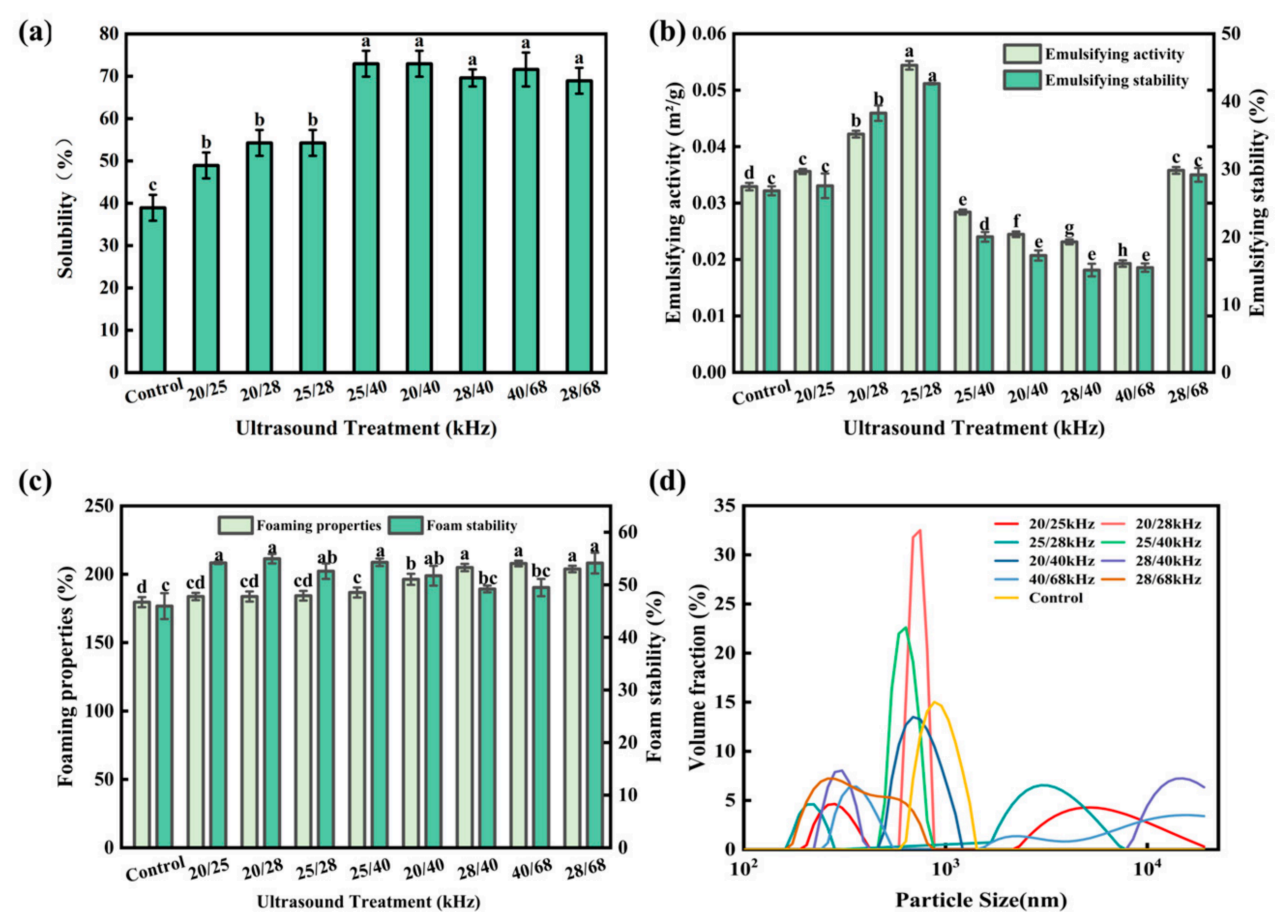
Effects of dual-frequency ultrasound-assisted glycation on the functional properties of beef MPs: (**a**) solubility, (**b**) emulsifying activity and emulsion stability, (**c**) foaming capacity and foam stability, and (**d**) particle size distribution of emulsions. Different lowercase letters indicate significant differences (*p* < 0.05).

Solubility is a key determinant of the functional attributes of proteins, as it directly affects their emulsification, gelation, foaming and water retention capacities within food systems [24]. As shown in Figure 3a, dual-frequency ultrasound-assisted glycation significantly improved the solubility of beef MPs compared with the control group. The control group showed the lowest solubility, whereas all ultrasound-treated groups exhibited higher values, indicating that the combined treatment effectively enhanced protein dispersion and hydration. Among the tested frequency combinations, the 25/40 and 20/40 kHz groups showed the highest numerical solubility values, followed by 40/68, 28/40, and 28/68 kHz, with no significant difference among these treatments. In contrast, the 20/25, 20/28, and 25/28 kHz groups showed only moderate increases in solubility. The enhanced solubility may be attributed to ultrasound-induced cavitation and mechanical shear. These effects promoted MP relaxation and unfolding, exposed more hydrophilic groups, and disrupted intermolecular hydrogen bonds and non-covalent interactions. The stronger improvement observed in the 20/40 and 25/40 kHz groups suggests that these frequency combinations provided a more suitable acoustic energy input for breaking protein aggregates and increasing protein–water interactions. By contrast, the relatively lower solubility in the 20/25, 20/28, and 25/28 kHz groups may be due to insufficient structural disruption, which limited the exposure of hydrophilic sites. Similar findings were reported by Lan et al. [25], who found that ultrasonication promoted protein unfolding and enhanced interactions between proteins and water molecules. This outcome is also consistent with the conclusions of Li et al. [26].

#### 2.3.2. Emulsifying Activity and Emulsion Stability

Emulsifying activity index (EAI) and emulsion stability index (ESI) are two crucial indicators for evaluating emulsification performance [27]. As shown in Figure 3b, dual-frequency ultrasound-assisted glycation affected the emulsifying properties of beef MPs in a frequency-dependent manner. The 25/28 kHz treatment exhibited the highest EAI and ESI values, followed by the 20/28 kHz treatment. These results indicated that the two frequency combinations were more favorable for improving interfacial adsorption and emulsion stability. The 20/25 and 28/68 kHz groups showed only slight improvement in EAI, whereas the 25/40, 20/40, 28/40, and 40/68 kHz treatments reduced both EAI and ESI compared with the control. The emulsifying activity of a protein is primarily determined by its behavior at the oil–water interface [28]. Therefore, the superior emulsifying performance of the 25/28 kHz group may be attributed to moderate protein unfolding, which optimized the hydrophilic–hydrophobic balance and promoted protein adsorption at the oil–water interface. Zhou et al. [29] also reported that ultrasound-induced conformational transitions could improve protein emulsifying performance by regulating the balance between hydrophilicity and hydrophobicity. In contrast, unsuitable frequency combinations may have induced excessive aggregation or unfavorable conformational rearrangement, thereby reducing the migration and adsorption capacity of MPs at the interface. Furthermore, Xiong et al. [30] demonstrated that increased structural ordering and self-assembly of proteins strengthen intermolecular interactions, consequently enhancing their interfacial adsorption and film-forming capabilities.

#### 2.3.3. Foaming Capacity and Foam Stability

Foaming capacity is an important indicator of the ability of proteins to form and maintain foam at the gas–liquid interface. Protein solubility is positively correlated with foaming ability; the more soluble the protein, the more effectively it can diffuse at the gas–liquid interface, thereby improving foaming performance [1]. According to Figure 3c, dual-frequency ultrasound-assisted glycation improved the foaming capacity of beef MPs in a frequency-dependent manner. The 28/40, 40/68, and 28/68 kHz groups exhibited the highest foaming capacity, followed by the 20/40 kHz group, whereas the 20/25, 20/28, and 25/28 kHz treatments showed only slight increases compared with the control. This outcome is in agreement with the findings of Fu et al. [31]. The higher foaming capacity observed in the 28/40, 40/68, and 28/68 kHz groups may be related to their stronger ability to unfold protein structures and expose surface-active groups, thereby facilitating protein diffusion and adsorption at the gas–liquid interface.

Foam stability is a key parameter for assessing a protein’s capacity to maintain foam structure. Han et al. [32] found that higher solubility of glycated MPs was associated with improved foam stability. As shown in Figure 3c, the 20/25, 20/28, 25/40, and 28/68 kHz groups showed the highest foam stability, while the 28/40 and 40/68 kHz groups exhibited relatively lower stability despite their higher foaming capacity. This suggests that the frequency combinations favorable for foam formation were not completely consistent with those beneficial for foam stabilization. This phenomenon might be due to the formation of a more cohesive film structure of glycated MPs at the air–water interface following ultrasonication [33]. Moderate ultrasound treatment may promote the formation of a flexible and cohesive interfacial film, whereas excessive unfolding or aggregation under some frequency combinations could weaken the interfacial film and reduce foam stability.

Overall, dual-frequency ultrasound-assisted glycation improved the foaming properties of beef MPs, but the effects varied depending on the frequency combination. The 28/40, 40/68, and 28/68 kHz treatments were more effective in enhancing foaming capacity, whereas 20/25, 20/28, 25/40, and 28/68 kHz were more favorable for maintaining foam stability. Therefore, the regulation of foaming behavior by ultrasound was not simply dependent on solubility, but also closely related to protein unfolding, interfacial adsorption, and film-forming ability.

### 2.4. Emulsion Properties of Beef MPs

#### 2.4.1. Particle Size Distribution

During the processing and storage of MPs, physicochemical interactions such as oxidation and denaturation can stimulate particle aggregation, thereby increasing the average particle size [34]. Ultrasonication has been shown to modulate the particle size distribution of MP emulsions. As illustrated in Figure 3d, all dual-frequency ultrasound-assisted glycation treatments altered the particle size distribution of beef MP emulsions, but the extent of change varied among different frequency combinations. Compared with the control, most ultrasound-treated samples showed a shift in the main particle size peak toward smaller sizes, indicating that ultrasound promoted the breakdown of large aggregates and increased the proportion of small- and medium-sized particles. This change may be ascribed to the mechanical disruption of large aggregates following ultrasonication, which also inhibited the reaggregation of particles, consequently reducing the average particle size. These observations are in line with those reported by Hong et al. [35].

The particle size distribution data in Table 2 further confirmed the frequency-dependent effect of dual-frequency ultrasound. The control group showed a small-particle peak at 636–1318 nm. In contrast, the main small-particle peaks of the ultrasound-treated groups shifted to lower ranges, including 204–391 nm for 20/25 kHz and 174–260 nm for 25/28 kHz. Among them, 25/28 and 28/68 kHz exhibited a more obvious shift toward smaller particle sizes, suggesting stronger dispersion effects. However, secondary large-particle peaks were observed in the 20/25, 25/28, 28/40, and 40/68 kHz groups, indicating that some frequency combinations may also induce partial reaggregation or droplet flocculation. This phenomenon may be associated with ultrasound-induced exposure and rearrangement of hydrophobic regions, which could enhance intermolecular hydrophobic interactions and thereby promote partial reaggregation or the formation of larger aggregates under certain frequency combinations [36]. Therefore, dual-frequency ultrasound did not simply reduce particle size uniformly; instead, its effect depended strongly on the specific frequency combination.

#### 2.4.2. Storage Stability

**Figure 4:**
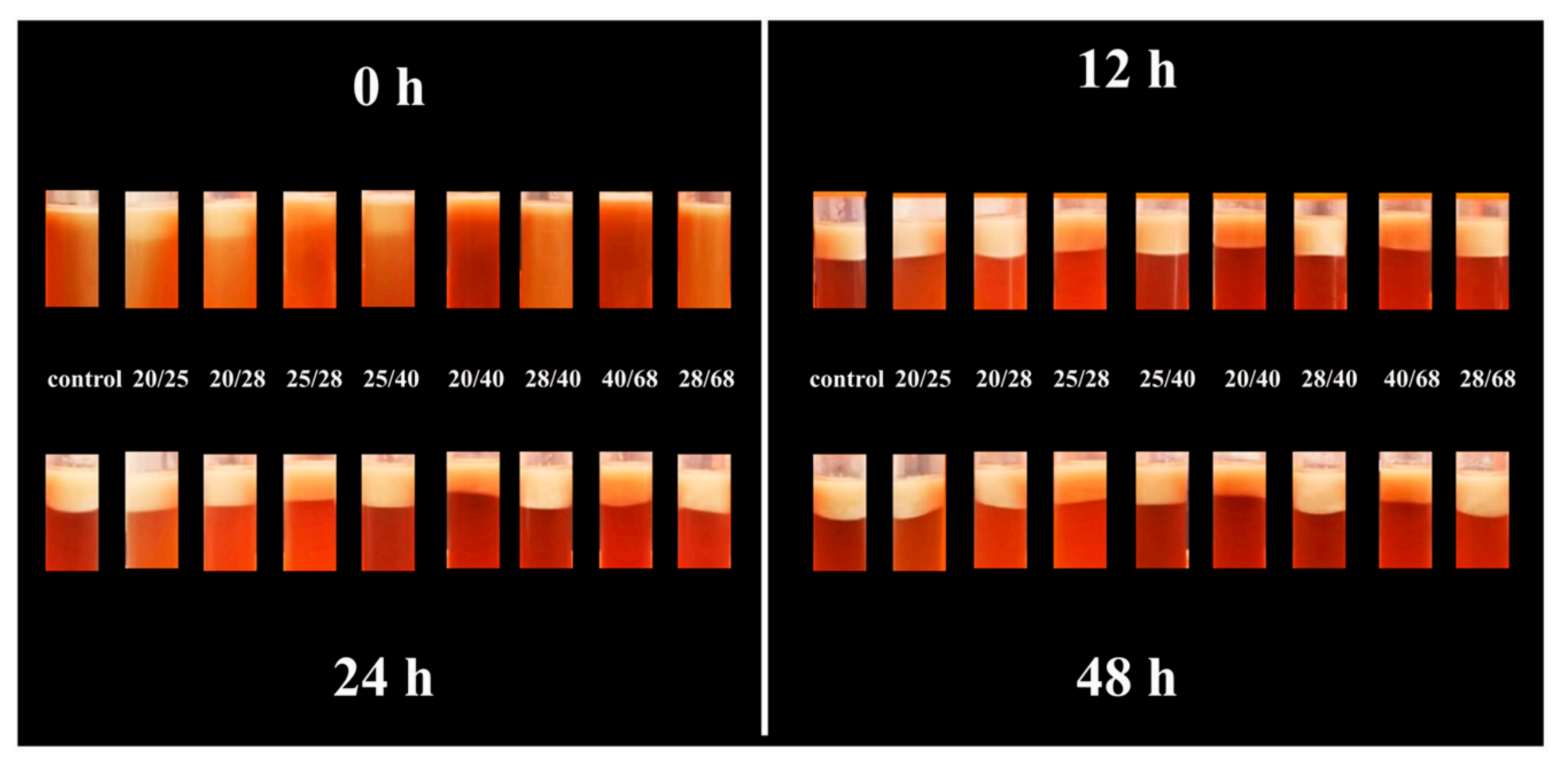
Representative photographs showing the storage stability of beef MP emulsions prepared after dual-frequency ultrasound-assisted glycation during storage for 0, 12, 24, and 48 h.

Storage stability indicates how well an emulsion resists droplet aggregation, whereas phase separation reflects the integrity of the emulsion structure. The significant phase separation phenomenon is usually negatively correlated with the storage stability of the emulsion [28]. Figure 4 shows the stratification variations of the nine MP emulsion groups during storage. The results indicate that although all samples exhibited varying degrees of phase separation, the overall stability was relatively stable. Most notably, the 25/40 kHz, 20/40 kHz, and 28/40 kHz groups showed more pronounced phase separation than the control, indicating poorer storage stability. This phenomenon suggests a possible threshold effect of ultrasound treatment. Moderate unfolding at 25/28 kHz may favor interfacial adsorption and protein film formation. In contrast, frequency combinations involving 40 kHz may induce excessive unfolding or aggregation, weakening the interfacial protein film and promoting phase separation. According to Weiss et al. [37], interfacial protein–lipid interactions are important for maintaining emulsion stability, because a stable interfacial layer can help protect dispersed oil droplets against aggregation.

### 2.5. Structural Properties

#### 2.5.1. Free Sulfhydryl Content

Sulfhydryl groups are active thiol moieties in protein molecules and are closely associated with the stability of the secondary structure of the protein. Upon protein unfolding, originally buried sulfhydryl groups become exposed, resulting in an increase in the content of the free sulfhydryl groups.

**Figure 5:**
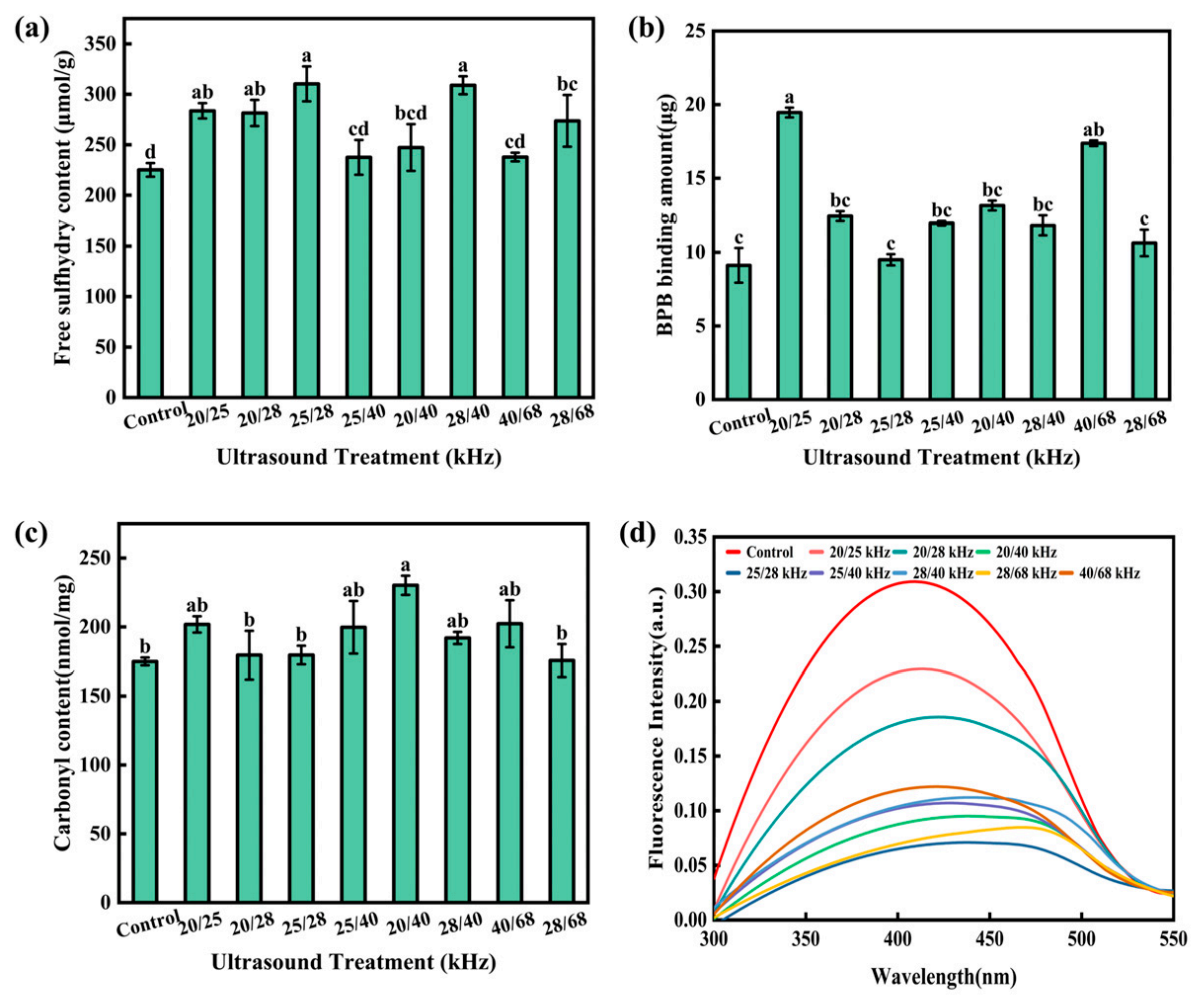
(**a**) free sulfhydryl content; (**b**) surface hydrophobicity; (**c**) carbonyl content; and (**d**) intrinsic fluorescence spectra. Different lowercase letters indicate significant differences among treatments (*p* < 0.05).

Figure 5a illustrates the variations in the free sulfhydryl group levels of MPs under different dual-frequency ultrasound conditions. Compared to the control, ultrasonication substantially improved the free thiol group content, indicating that ultrasound promoted the exposure of thiol groups in proteins. Comparable results have also been reported by Chen et al. [38]. However, Xu et al. [14] found that excessive exposure of free sulfhydryl groups may affect the reactivity of lysine residues through the steric hindrance effect.

Among all dual-frequency combinations, the 25/28 kHz and 28/40 kHz treatment groups showed the highest free sulfhydryl content, indicating that ultrasound promoted the exposure of originally buried thiol groups. However, the functional implication of thiol exposure differed between these two treatments. The 25/28 kHz group combined high free sulfhydryl content with relatively low carbonyl content and improved gel properties, suggesting that thiol exposure was associated with favorable network formation. By contrast, the 28/40 kHz group also showed high free sulfhydryl content but had elevated carbonyl content (Figure 5c), indicating stronger oxidative modification. This explained why the increased free sulfhydryl content in this group did not necessarily translate into superior gelation performance. According to the interface effect theory proposed by Ma et al. [39], high-frequency ultrasound may facilitate the migration of exposed groups to the protein network surface. Intermolecular interactions at this interface may then promote reoxidation and the formation of intra- and intermolecular disulfide bonds.

#### 2.5.2. Surface Hydrophobicity

Surface hydrophobicity is a crucial indicator of the ability of the protein to repel water and thus reflects the physicochemical characteristics of its surface molecular network [14]. As presented in Figure 5b, under different dual-frequency ultrasound conditions, the surface hydrophobicity of most sonicated groups was improved to varying degrees. Among all treatments, the 20/25 kHz group exhibited the highest BPB binding amount, followed by the 40/68 kHz group, indicating that these two frequency combinations were more effective in exposing hydrophobic groups. The 20/28, 25/40, 20/40, and 28/40 kHz groups showed moderate increases, whereas the 25/28 and 28/68 kHz groups showed relatively low values close to the control. This increase may be attributed to the rearrangement of the protein molecular configuration caused by the cavitation effect of ultrasonication, which exposes hydrophobic amino acid residues originally buried inside the protein molecule, thus increasing the surface hydrophobicity [40]. However, the changes in surface hydrophobicity did not follow a simple frequency-dependent trend, suggesting that the exposure of hydrophobic residues was closely related to the specific dual-frequency combination rather than ultrasound frequency alone. Under the four frequency combinations of 25/28 kHz, 25/40 kHz, 28/40 kHz, and 28/68 kHz, surface hydrophobicity initially increased and then decreased with increasing frequency. This may be explained by the fact that higher ultrasonic frequency could intensify cavitation and mechanical action, causing partial unfolding of protein and, thus, exposure of hydrophobic groups [41]. Nonetheless, under some treatments, excessive unfolding or re-aggregation of exposed hydrophobic regions may cause hydrophobic groups to be buried again, thus reducing surface hydrophobicity [42].

#### 2.5.3. Carbonyl Content

During protein glycation, molecules are prone to cross-linking and aggregation, leading to the conversion of some amino acid residues into carbonyl compounds. This process subsequently impairs or even abolishes the biological function of the proteins. As shown in Figure 5c, dual-frequency ultrasound-assisted glycation increased the carbonyl content of beef MPs to different extents, depending on the frequency combination. Among all treatments, the 20/40 kHz group showed the highest carbonyl content, indicating the strongest protein oxidation under this condition. The 20/25, 25/40, 28/40, and 40/68 kHz groups also showed relatively high carbonyl contents, whereas the 20/28, 25/28, and 28/68 kHz groups were close to the control. This increase reflects the extent of oxidative degradation of amino acid residues [43], demonstrating that ultrasound-assisted treatment could intensify protein oxidation during the glycation reaction. The higher carbonyl content observed at 20/40 kHz may be attributed to stronger cavitation and shear effects, which promoted protein unfolding and exposed more oxidation-sensitive amino acid residues. However, under some frequency combinations, such as 20/28, 25/28, and 28/68 kHz, the relatively lower carbonyl content may be related to milder structural disruption or partial aggregation, which limited the exposure of oxidation-prone sites [25]. Combined with the results of Figure 1, the increase in carbonyl content partly corresponded to the enhancement of DG. This indicates that glycation and oxidative modification may occur concurrently during ultrasound-assisted alkaline glycation. Moderate oxidation may promote protein unfolding and expose reactive sites, but excessive oxidation may also contribute to amino group loss or protein aggregation and should be distinguished from Maillard grafting.

#### 2.5.4. Intrinsic Fluorescence Spectroscopy

Intrinsic fluorescence spectroscopy exploits the sensitivity of tryptophan residues to alterations in the microenvironment, which can be used as an indirect means of evaluating changes in protein conformation. According to Figure 5d, the control group had higher fluorescence intensity than all ultrasound-treated groups, which is consistent with the results of Xu et al. [14] on protein oxidation and folding. This indicates that ultrasound-induced aggregation and conformation rearrangement may have modified the spatial arrangement of the protein, resulting in the oxidation of tryptophan residues, and consequently, a reduction in fluorescence intensity. This finding shows that ultrasound treatment was effective in altering the tertiary structure of proteins. Moreover, the samples treated with 20/25, 20/28 and 40/68 kHz showed relatively higher fluorescence intensities. This may be attributed to the mechanical shear forces generated by these specific frequency combinations, which preferentially disrupted secondary molecular bonds, promoted the partial decomposition of the protein, and exposed originally buried hydrophobic groups [44]. Compared with Figure 5b, the surface hydrophobicity of these three sample groups was found to be higher, further corroborating the increased exposure of hydrophobic residues.

#### 2.5.5. Fourier Transform Infrared Spectroscopy (FTIR)

**Figure 6:**
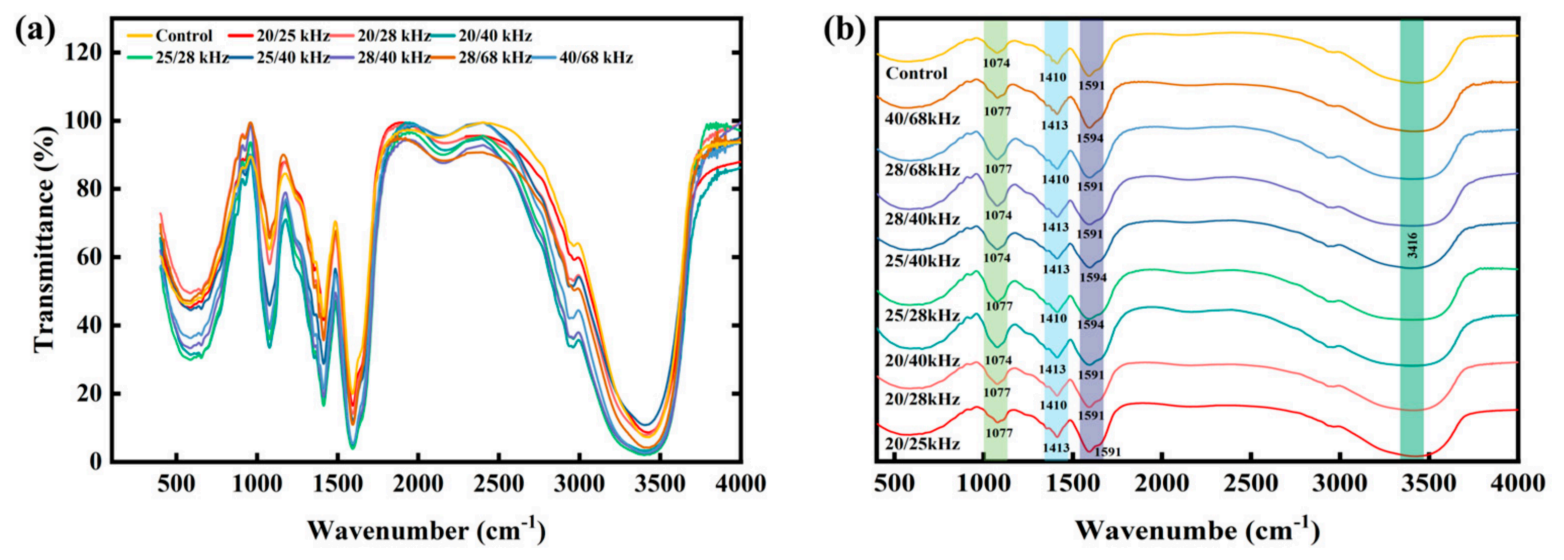
Fourier transform infrared (FTIR) spectra of beef MPs subjected to dual-frequency ultrasound-assisted glycation: (**a**) overlaid spectra; (**b**) vertically offset spectra with characteristic peaks highlighted.

Figure 6 presents the infrared spectral characteristics of beef MPs treated with different dual-frequency ultrasound combinations. Following glycation modification, distinct absorption peaks appeared in the range of 1050–1000 cm^−1^, which correspond to the symmetric stretching vibration of C–O–C glycosidic bonds and hydroxyl groups [45]. This shift in the amide A band (around 3416 cm^−1^) reflects the changes in the expansion and stretching vibration modes of O–H, N–H, and C–H bonds, indicating the formation of a new intermolecular hydrogen bond network during the glycation process. Compared to the control group, the characteristic peaks of MP conjugates treated with dual-frequency ultrasound treatment were enhanced as a whole, of which the 25/28 kHz, 28/40 kHz, and 40/68 kHz treatment groups were the most significant. These spectral changes show that the mechanical shear forces generated by ultrasonication may have destroyed the advanced structure of proteins [42], promoting the unfolding of the molecular structure and exposure of reactive sites, thus improving the efficiency of carbohydrate conjugation.

Further analysis of the characteristic amide bands revealed significant changes in the C=O stretching vibration of the amide I band (1700–1600 cm^−1^), the N–H bending vibration and C–N stretching vibration of the amide II band (1574–1480 cm^−1^), and the C–H stretching vibration of the amide III band (1400–1200 cm^−1^). Following ultrasonication, the absorption peak intensity in the amide region markedly increased, indicating protein unfolding and consequent exposure of additional amino groups, thus promoting the glycation reaction. This outcome is consistent with the findings of Liu et al. [42].

#### 2.5.6. Scanning Electron Microscopy (SEM)

**Figure 7:**
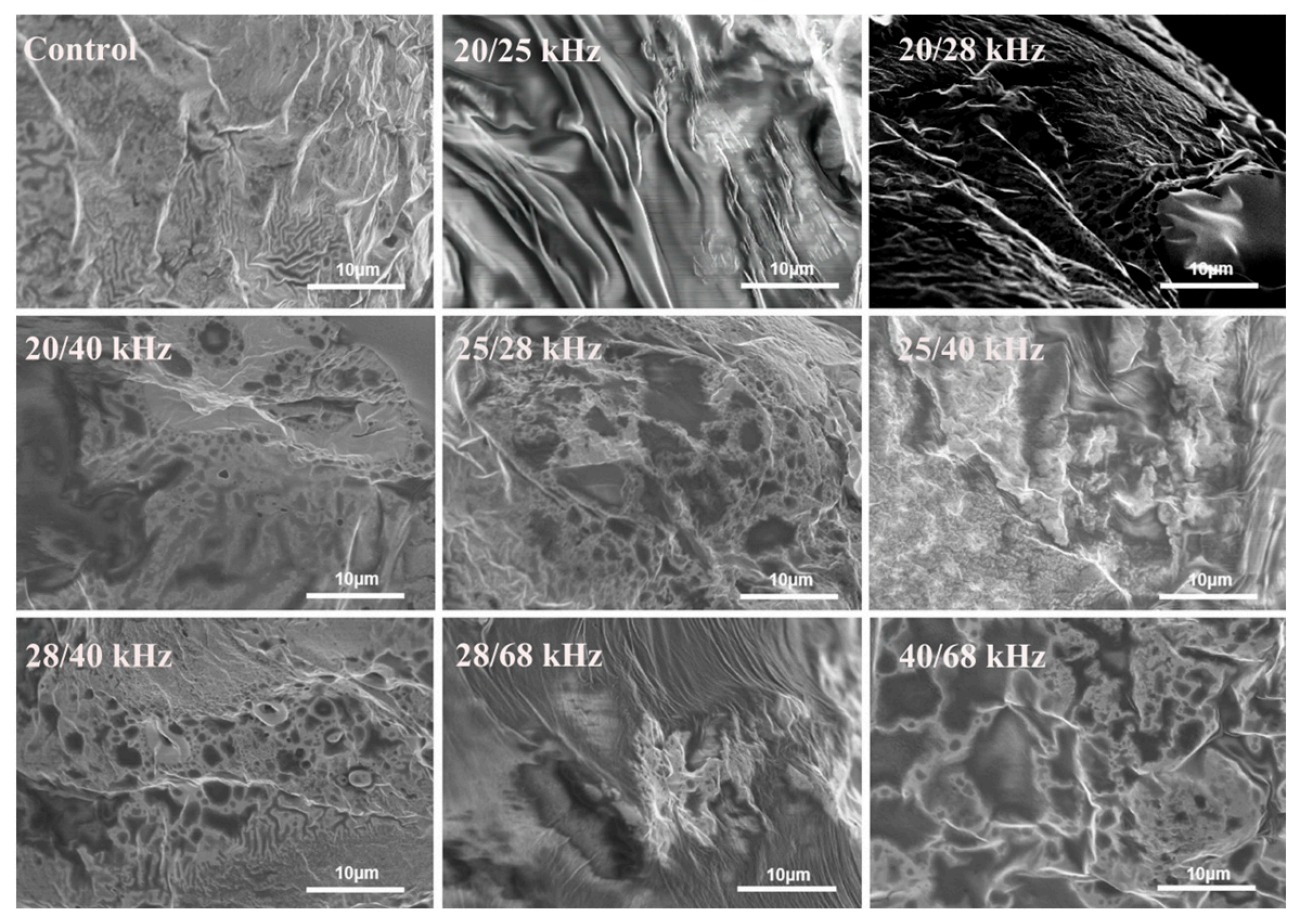
Scanning electron microscopy (SEM) images of beef MPs after dual-frequency ultrasound-assisted glycation. From (**left**) to (**right**) and (**top**) to (**bottom**), the images correspond to the control, 20/25, 20/28, 20/40, 25/28, 25/40, 28/40, 28/68, and 40/68 kHz treatments, respectively. Scale bars = 10 μm.

Figure 7 shows the effects of various dual-frequency ultrasound treatments on the surface morphology of MPs. According to Wang et al. [45], the surface of MPs usually becomes rougher following the grafting reaction with xylose. The findings of this study agree with this conclusion. This effect may be linked to the moderate molecular weight of the xylose used, which prevented the formation of smooth macromolecular polymers on the protein surface.

From a micromorphological perspective, the surface of the samples in the control group was slightly curled, whereas those subjected to dual-frequency ultrasound displayed various degrees of surface cavitation. This phenomenon is mainly attributed to the cavitation effect generated by ultrasound, which induced localized structural perturbations in the protein. Upon collapse of the cavitation bubbles, the instantaneous release of energy promotes intramolecular aggregation or crosslinking of proteins [42]. Among the frequency combinations, the 25/28 kHz, 25/40 kHz, and 40/68 kHz treatments caused the most pronounced effects on protein structure, characterized by more extensive and distinct cavitation features. This indicates that these specific frequency combinations generated stronger cavitation intensity, thereby exerting a more substantial regulatory and modifying effect on the three-dimensional conformation of the proteins.

### 2.6. LAL Content

**Figure 8:**
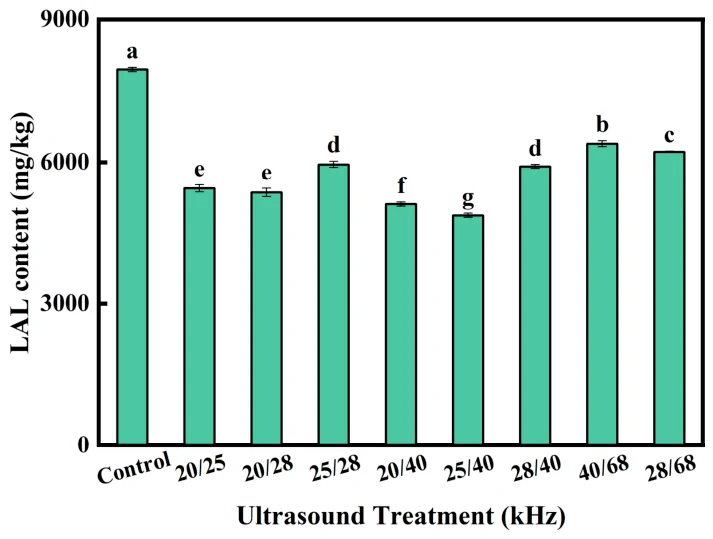
Effects of dual-frequency ultrasound-assisted glycation on LAL content in beef MPs. Different lowercase letters indicate significant differences (*p* < 0.05).

**Figure 9:**
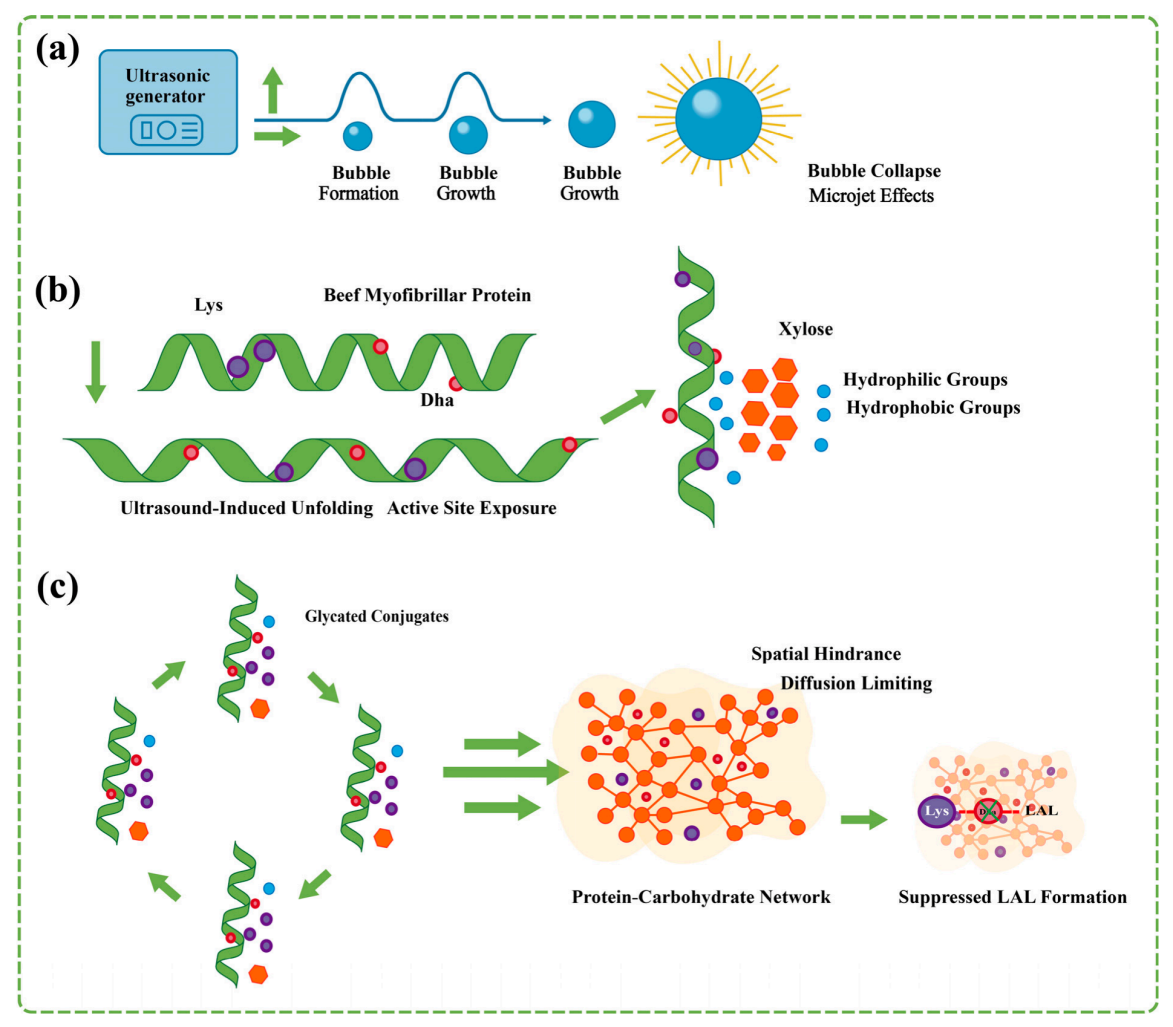
Mechanism diagram of dual-frequency ultrasound-assisted glycation suppressing LAL formation in beef MPs. (**a**) Ultrasound-induced cavitation, bubble growth and collapse, and microjet effects; (**b**) ultrasound-induced MP unfolding, exposure of Lys- and Dha-related reactive sites, and interaction with xylose; (**c**) formation of glycated protein–carbohydrate networks, resulting in steric hindrance and diffusion limitation and thereby suppressing LAL formation.

Figure 8 shows the changes in LAL content in beef treated with dual-frequency ultrasound-assisted glycation. Relative to the control, all dual-frequency ultrasound treatments markedly reduced the LAL content in the samples. Specifically, the LAL content decreased from 7961.24 mg/kg in the control to 4868.88 mg/kg in the 25/40 kHz group, corresponding to a reduction of approximately 38.8%. Among these, the sample treated with 25/40 kHz exhibited the lowest LAL content, followed by the 20/40 kHz group. Moderate inhibition effects on LAL formation were observed in the 25/28 kHz, 20/28 kHz and 28/40 kHz groups, whereas the 40/68 kHz and 28/68 kHz combinations exhibited the weakest inhibition effects. Previous studies have reported that LAL can occur in processed protein foods and may be used as an indicator of alkali- or heat-induced protein damage and reduced nutritional quality. However, direct comparison should be made cautiously because LAL levels are strongly affected by food matrix, processing conditions, and reporting units [46,47]. Figure 9 illustrates the proposed mechanism underlying this phenomenon. The cavitation and microjet effects induced by sonication stimulated protein conformational rearrangement and redistribution of carbohydrate molecules, thereby promoting the formation of a denser protein–carbohydrate network structure. The resulting spatial hindrance and diffusion-limiting effects reduced the contact between precursors of the Dha pathway and lysine residues, thus effectively inhibiting LAL formation. Similar mechanisms have been corroborated by the reports of Xu et al. [15].

In previous studies, MPs treated with 25/28 kHz ultrasound exhibited excellent functional, structural, and emulsifying properties. However, in the present experiment, its inhibitory effect on LAL formation was not the most pronounced. This may be attributed to the relatively mild nature of this frequency combination. Although it moderately promoted protein unfolding and interfacial adsorption, it failed to induce pronounced noncovalent aggregation or steric barrier structures, thereby limiting its capacity to markedly reduce LAL formation. In contrast, the 25/40 kHz and 20/40 kHz treatments, operating under stronger acoustic energy fields, triggered more extensive conformational rearrangement and molecular aggregation. This may have enhanced the spatial restriction within the system and consequently decreased the probability of cross-linking between lysine residues and dehydroalanine intermediates/precursors. Comparable outcomes have also been reported by Gräfenhahn and Beyrer [48].

## 3. Conclusions

Dual-frequency ultrasound-assisted glycation regulated beef MP properties through coupled effects on Maillard conjugation, protein unfolding, oxidative modification, interfacial adsorption, gel network formation, and LAL generation. These responses were frequency-dependent and were not determined by a single quality or safety indicator. Among the tested treatments, 25/28 kHz showed the most balanced performance for gel-type applications, probably because moderate protein unfolding and protein–xylose conjugation promoted a compact water-retaining gel network and favorable interfacial behavior while avoiding excessive oxidative modification. In contrast, 25/40 kHz was more effective in suppressing LAL formation, suggesting that stronger structural rearrangement may enhance steric restriction around LAL-related precursor reactions; however, this did not translate into superior overall gel quality. Therefore, functional enhancement and LAL inhibition were partially decoupled during alkaline ultrasound-assisted glycation, and frequency selection should be target-oriented rather than based on a single response. Overall, 25/28 kHz may be more suitable when balanced gel quality, emulsifying performance, and safety control are required, whereas 25/40 kHz may be preferred when LAL reduction is the primary objective. This study provides a frequency selection basis for applying dual-frequency ultrasound-assisted glycation to improve the quality and safety of beef MP-based gel products. Nevertheless, this study was conducted in a controlled MP–xylose alkaline glycation system, and sensory quality, digestive behavior, and molecular-level interactions related to LAL inhibition were not directly evaluated. Future studies should validate the selected frequency conditions in real meat product systems and combine sensory evaluation, in vitro digestion, and molecular simulation to further clarify the quality and safety implications of this modification strategy.

## 4. Materials and Methods

### 4.1. Sample Preparation

Beef hind leg meat (24-month-old yellow cattle, supplied by Shandong Huasheng Halal Meat Co., Ltd., Binzhou, Shandong, China) was trimmed immediately after slaughtering to remove the fascia and visible fat layers. The meat was then aged for 72 h and subsequently cut into cubes (1 cm^3^). The prepared samples were divided into 9 treatment groups. Three independent experimental batches were included, and each batch contained all treatment groups to ensure biological and experimental reproducibility. Unless otherwise stated, all reagents used for chemical analyses were analytical grade and purchased from Sinopharm Chemical Reagent Co., Ltd. (Shanghai, China).

### 4.2. Extraction of MPs

MPs were extracted following the approach of Zhao et al. [49] with minor modifications. Beef samples were homogenized with phosphate-buffered saline (PBS; 0.01 mol/L, pH 7.2), and the non-myofiber components were removed through repeated washing and centrifugation, purified with 0.1 mol/L NaCl solution, and then kept at 4 °C until further use.

### 4.3. Dual-Frequency Ultrasound-Assisted Glycation

The ultrasonic treatment method was performed as previously detailed by Shi et al. [50] with some modifications. The MP precipitate was dispersed in a 3% xylose solution (Sinopharm Chemical Reagent Co., Ltd., Shanghai, China) at ratio of 1:20 (*w*/*v*). The pH of each treatment group was then adjusted to 12.5, using a 2 mol/L NaOH solution. The grafting reaction was initiated at 80 °C under dual-frequency ultrasonication at a power of 480 W, using a pulse mode of 10 s on/5 s off, for 20 min. Owing to the fixed transducer arrangement and control configuration of the laboratory-built five-frequency ultrasound system, stable simultaneous operation was available in eight dual-frequency modes: 20/25, 20/28, 20/40, 25/28, 25/40, 28/40, 28/68, and 40/68 kHz. These reproducible operating modes covered different frequency levels and frequency separations and were therefore selected for comparison. The reaction was subsequently maintained at 80 °C with magnetic stirring for 100 min, ensuring a total reaction time of 2 h. MP samples with xylose addition and the same pH shift, heating temperature, and reaction time, but without ultrasonic treatment, were used as non-ultrasonicated glycated controls. Following the completion of all reactions, the samples were cooled to 25 °C, neutralized to pH 7.0, kept at −20 °C for 24 h, and finally transferred to −80 °C for storage.

### 4.4. Grafting Degree

The protocol of Pan et al. [51] was followed to determine the grafting degree (DG) of MP–xylose conjugates, and the free amino group content was determined using O-phthalaldehyde by measuring the absorbance at 340 nm. The DG was calculated as follows:

(1) $$DG(\%)=\frac{\left(A_{1}-A_{2}\right)}{A_{1}}\times 100\%$$

where *DG* (%) represents the grafting degree, *A*_1_ represents the absorbance of the sample before grafting, and *A*_2_ represents the absorbance of the sample after grafting.

### 4.5. Gel Preparation and Analysis

#### 4.5.1. Gel Preparation

Gel was prepared according to the method described by Liu et al. [5] with slight modifications. The treated myofibrillar protein was dissolved in 0.6 mol/L NaCl and 0.5 mol/L phosphate buffer, and the pH was adjusted to 6.2–6.5 to obtain a protein solution at a final concentration of 40 mg/mL. The solution was homogenized at 10,000 r/min for 1 min to ensure uniform protein dispersion. After homogenization, the samples were allowed to stand for 10 min to remove entrapped air bubbles. The samples were then heated in a water bath at 40 °C for 30 min, followed by a second heating step at 90 °C for 30 min. Afterward, the samples were immediately cooled in an ice bath to room temperature and stored at 4 °C overnight to allow complete gel formation.

#### 4.5.2. Color Measurement

The color of the gels was measured using a chroma meter (CR-400, Konica Minolta, Tokyo, Japan). The L* (lightness), a* (redness/greenness), and b* (yellowness/blueness) values were recorded. The total color difference (Δ*E*) relative to the control was calculated as follows:

(2) $$\Delta E=\sqrt{{\left(L^{*}-{L_{0}}^{*}\right)}^{2}+{\left(a^{*}-{a_{0}}^{*}\right)}^{2}+{\left(b^{*}-{b_{0}}^{*}\right)}^{2}}$$

where ${L_{0}}^{*}$, ${a_{0}}^{*}$, and ${b_{0}}^{*}$ represent the mean color coordinates of the control sample.

#### 4.5.3. Cooking Loss

Cooking loss was quantified based on the weight change of samples before and after thermal gelation. Briefly, the protein solution was weighed prior to heating (*m*_1_), then heated sequentially at 40 °C for 30 min and 90 °C for 30 min. After cooling to room temperature in an ice bath, the released surface moisture was removed, and the resulting gel was weighed again (*m*_2_). Cooking loss was calculated as follows:

(3) $$Cooking\;loss(\%)=\frac{m_{1}-m_{2}}{m_{1}}\times 100\%$$

#### 4.5.4. Texture Properties

The gel texture was assessed using a texture analyzer (TA.XTPLUSC, Stable Micro Systems, Godalming, Surrey, UK) following the method of Tan et al. [52] with slight modifications. A cylindrical probe (P/36R) was used for a two-cycle compression test. The pre-test, test, and post-test speeds were all set to 1.0 mm/s, and the compression distance was 50% of the original height of the sample.

#### 4.5.5. Dynamic Rheological Properties

The dynamic rheological properties of the formed MP–xylose gels were evaluated according to the method of Wang et al. [53] using a rheometer (HAAKE MARS II, Thermo Fisher Scientific, Karlsruhe, Germany). After gel preparation as described in Section 4.5.1, the gel samples were equilibrated to room temperature and carefully loaded onto the rheometer equipped with parallel plates (35 mm diameter). The measurement gap was set to 1.0 mm, and a thin layer of silicone oil was applied around the edges of the samples to prevent moisture loss during testing. A frequency sweep was performed over a range of 0.1 to 100 Hz at a constant strain of 1%, and the storage modulus (G′), loss modulus (G″), and loss factor (tan δ) were recorded.

### 4.6. Functional Properties

#### 4.6.1. Solubility

Using the Li et al. [54] approach, but with some changes, protein solubility was determined. Samples were dissolved in PBS (10 mg/mL) containing 0.1 mol/L NaCl. The samples were then centrifuged at 10,000× *g* at 4 °C for 15 min, and the protein concentration in the supernatant was quantified. Solubility was computed as follows:

(4) $$PS(\%)=\frac{Cs}{Ci}\times 100\%$$

where *PS* (%) represents the protein solubility, *C_s_* represents the protein concentration in the supernatant, and *C_i_* represents the initial protein concentration.

#### 4.6.2. Emulsion Property

MP samples were dissolved in PBS to obtain a final concentration of 4 mg/mL. The emulsion was formed by blending 8 mL of the diluted MP solution with 2 mL of soybean oil (Fulinmen, COFCO Group, Beijing, China) and homogenizing at 15,000 r/min for 60 s using a homogenizer (FSH-2A, Jiangsu Jinyi Instrument Technology Co., Ltd., Changzhou, China). To evaluate emulsion stability, at 0 min and 30 min after emulsion preparation, 6 μL samples were taken from the bottom layer of the emulsion using a micropipette (DLAB Scientific Co., Ltd., Beijing, China). Each sample was immediately diluted 500-fold with 3 mL of 0.1% sodium dodecyl sulfate (SDS; Beyotime Biotechnology Co., Ltd., Shanghai, China) solution. The SDS solution without protein served as a blank control. The absorbance values at 0 min (*A*_0_) and 30 min (*A*_30_) were measured using a UV–visible spectrophotometer (UV-600, Shanghai Meipuda Instrument Co., Ltd., Shanghai, China). The emulsion activity (EAI) and emulsion stability (ESI) were computed as follows:

(5) $$EAI(m^{2}/g)=\frac{2.303\times 2\times A_{0}\times N}{c\times \varphi \times 10000}$$

(6) $$ESI(\min)=\frac{A_{0}}{A_{0}-A_{30}}\times 10$$

where *A*_0_ is the absorbance value of the diluted emulsion measured immediately after formation, *A*_30_ is the absorbance value of the diluted emulsion after standing for 30 min; *N* is the dilution factor of 500, *c* is the protein concentration (g/mL); and *φ* represents the volume fraction of soybean oil at 0.2.

#### 4.6.3. Foaming Property

Following the procedure described by Wang et al. [55], the foaming properties were evaluated. MPs were dissolved in PBS at a concentration of 10 mg/mL. A 10 mL aliquot of sample solution was homogenized at 18,000 r/min for 2 min, and the foam volume was recorded at 0 and 30 min, respectively. The foaming ability (FA) and foam stability (FS) were subsequently calculated as follows

(7) $$FA(\%)=\frac{V_{0}}{V}\times 100\%$$

(8) $$FS(\%)=\frac{V_{30}}{V_{0}}\times 100\%$$

where *V* denotes the volume of the sample solution (10 mL), *V*_0_ denotes the volume of the sample immediately after homogenization (mL), and *V*_30_ denotes the volume of the sample after standing for 30 min (mL).

### 4.7. Preparation of Emulsion of MPs

The samples were dissolved in PBS to prepare a suspension at a concentration of 8 mg/mL. An oil-in-water emulsion was prepared by mixing 4 mL of the MP suspension with 1 mL of soybean oil, resulting in a 20% oil phase. The mixture was homogenized at 10,000 r/min for 30 s in an ice-water bath. The freshly prepared emulsions were immediately kept at 4 °C, and all subsequent analyses were performed within 12 h of preparation.

#### 4.7.1. Particle Size Distribution

To analyze the particle size distribution of the emulsions, fresh emulsion samples prepared from an 8 mg/mL MP suspension with 20% soybean oil phase were diluted 1000-fold with 0.1% (*w*/*v*) sodium dodecyl sulfate solution. An aliquot of each diluted sample (1 mL) was transferred into a quartz cuvette, and the particle size and size distribution were determined using dynamic light scattering.

#### 4.7.2. Storage Stability

The storage stability of the emulsion systems was evaluated using an accelerated stability method. Nine sets of samples were stored at 4 °C for varying durations (0 h, 12 h, 24 h, and 48 h). During the storage period, the lipid separation in the emulsions was periodically examined and documented using a digital camera (Canon Inc., Tokyo, Japan, model RF50), allowing the assessment of their storage stability.

### 4.8. Structural Characteristics

#### 4.8.1. Free Sulfhydryl Content

A 76.25 mL aliquot of 0.2 mol/L Na_2_HPO_4_ solution and 48.75 mL of 0.2 mol/L NaH_2_PO_4_ solution were mixed in a beaker. Then, 1.681 g EDTA and 22.365 g KCl were added, followed by deionized water to reach a total volume of 500 mL.

A 1 mL aliquot of the sample prepared in Section 4.3 was mixed with 9.0 mL of the above-prepared solution and stirred for 1 h. Afterward, a 4 mL aliquot of the resultant was added to 0.4 mL of 0.1% DTNB solution. The mixture was allowed to react at 25 °C for 25 min. A PBS-DTNB mixture without sample was used as the blank control. The absorbance was read at 412 nm using a UV–visible spectrophotometer (Shanghai Mapada Instruments Co., Ltd., Shanghai, China).

(9) $$C_{0}=\frac{A_{412}\times D\times 10^{6}}{e}$$

where *C*_0_ (μmol/g) represents the free thiol content, *A*_412_ is the absorbance value, e is the extinction coefficient, and *D* is the dilution factor.

#### 4.8.2. Surface Hydrophobicity

Surface hydrophobicity of beef MP–xylose conjugates was examined as previously outlined by Wang et al. [55]. The sample was dispersed in PBS (0.01 mol/L, pH 7.2) to a concentration of 2 mg/mL. Then, 80 μL of 1 mg/mL bromophenol blue (BPB) solution was added to the 2 mL sample. The resulting mixture was left to react at 25 °C for 20 min and then centrifuged at 4000× *g* for 15 min. Thereafter, the supernatant was diluted 10-fold, and the absorbance was measured at 595 nm using a microplate reader (Infinite 200 PRO, Tecan Group Ltd., Männedorf, Switzerland). PBS was used as the blank. The surface hydrophobicity was calculated as follows:

(10) $$B_{0}=\frac{A_{0}-A_{S}}{A_{0}}$$

where *B*_0_ (μg) represents the binding amount of bromophenol blue. *A*_0_ and *A_s_* represent the absorbance values at 595 nm for the control and sample, respectively.

#### 4.8.3. Carbonyl Content

Protein carbonyl content was investigated using the colorimetric method with DNPH. A 0.1 mL of MP sample solution was suspended in 0.5 mL of HCl solution (containing 2,4-dinitrophenylhydrazine). After 25 min of reaction, trichloroacetic acid was added, vortexed, and then centrifuged at 10,000× *g* for 10 min. An equal volume of 2 mol/L HCl solution was used as the blank control. The protein precipitate was washed three times with an ethanol–ethyl acetate solution, suspended in guanidine hydrochloride solution, and then incubated in a water bath at 37 °C for 30 min. The absorbance was measured at 370 nm using a UV–visible spectrophotometer. The carbonyl content was determined as follows:

(11) $$C_{C}=\frac{A\times n\times 1000}{C\times D\times \varepsilon }$$

where *C_c_* is the carbonyl content, *A* is the blank-corrected absorbance, *C* (mg/mL) is the protein concentration, *D* (cm) is the path length, *ε* is the molar extinction coefficient of the DNPH-carbonyl complex, and *n* is the dilution factor.

#### 4.8.4. Intrinsic Fluorescence Spectroscopy

A 10 mg portion of MP–xylose conjugate was accurately weighed and dissolved in 10 mL PBS. The mixture was vortexed for 5 min to ensure complete dispersion and centrifuged at 2250× *g* for 15 min. The intrinsic fluorescence spectra of the samples were recorded using an F-320 fluorescence spectrophotometer (Beijing Rayleigh Analytical Instrument Corp., Beijing, China) at an excitation wavelength of 281 nm, with the emission spectra collected over the range of 300–450 nm. The excitation and emission slit widths were both set at 10 nm, and the scanning speed was 20 nm/s. An equal volume of PBS buffer served as the blank control.

#### 4.8.5. Fourier Transform Infrared Spectroscopy (FTIR)

The FTIR spectral characteristics of MP–carbohydrate conjugates were examined using Attenuated Total Reflection Fourier Transform Infrared Spectroscopy (670-IR + 610-IR, Agilent Technologies Inc., Santa Clara, CA, USA). The spectra were acquired at a wavenumber range of 4000–600 cm^−1^, a resolution of 4 cm^−1^, with 32 scans to improve the signal-to-noise ratio. The amide I region (1700–1600 cm^−1^) was processed using Omnic 9.2 (Thermo Fisher Scientific, Waltham, MA, USA) and PeakFit 4.12 (Systat Software Inc., San Jose, CA, USA) to assist spectral assignment and qualitative comparison of protein structural changes.

#### 4.8.6. Scanning Electron Microscopy (SEM)

Double-sided adhesive tape was used to secure the freeze-dried MPs onto circular metal stubs. The samples were then gold-sputter-coated with a Mini Sputter Coater (SC7620, Quorum Technologies, Laughton, UK) prior to imaging with a scanning electron microscope (S4800, Hitachi, Tokyo, Japan), as described by Zhang et al. [45].

### 4.9. LAL Content

The method of Li et al. [13] was followed to quantify the LAL content, using high-performance liquid chromatography (HPLC). A 0.10 g portion of freeze-dried MPs was dissolved in 10 mL of HCl solution (6 mol/L). Following concentration in a headspace vial, the sample was reconstituted in borate buffer. For a derivatization reaction in the dark, an equal volume of dansyl chloride solution was added. The HPLC analysis was conducted using a Kromasil RP-18 column (5 μm, 250 mm × 4.6 mm) coupled to a UV detector (Shimadzu Corporation, Kyoto, Japan) operated at 254 nm. Mobile phase A was 10 mmol/L PBS (pH 7.0), and mobile phase B was acetonitrile. The flow rate was 1.0 mL/min, with the following gradient program: 30–45% B from 0 to 15 min, 45–75% B from 15 to 17 min, 75% B from 17 to 20 min, 75–30% B from 20 to 23 min, and 30% B from 23 to 30 min.

### 4.10. Statistical Analysis

A one-way ANOVA was conducted using SPSS 25.0 software. Significant differences among mean values were analyzed using the Waller–Duncan multiple comparison test at *p* < 0.05. Graphs were plotted using Origin 2024. The results are presented as the mean ± SD of at least three independent experiments. Unless otherwise stated, each analytical determination was performed in triplicate using independently prepared samples. For qualitative observations, such as emulsion appearance and SEM, representative images were selected from repeated observations.

## Figures and Tables

**Table 1:** Color difference, cooking loss and texture properties of myofibrillar protein gels prepared by ultrasound-assisted glycation.

Treatment	L*	a*	b*	Δ*E*	Cooking Loss (%)	Hardness (g)	Springiness	Cohesiveness	Chewiness (g)	Resilience
Control	75.90 ± 0.45 ^a^	2.42 ± 0.09 ^a^	1.18 ± 0.22 ^a^	-	79.80 ± 1.23 ^bc^	1388.18 ± 18.19 ^b^	0.872 ± 0.011 ^ab^	0.556 ± 0.008 ^b^	673.03 ± 15.61 ^cde^	0.215 ± 0.007 ^a^
20/25 kHz	76.84 ± 0.48 ^a^	2.21 ± 0.08 ^a^	1.65 ± 0.27 ^a^	1.08 ± 0.51 ^a^	78.17 ± 0.74 ^cd^	1671.85 ± 163.40 ^a^	0.865 ± 0.058 ^ab^	0.832 ± 0.013 ^a^	1203.20 ± 144.08 ^a^	0.321 ± 0.148 ^a^
20/28 kHz	76.90 ± 0.60 ^a^	2.28 ± 0.16 ^a^	1.44 ± 0.42 ^a^	1.07 ± 0.69 ^a^	85.10 ± 2.00 ^a^	1417.13 ± 81.91 ^b^	0.896 ± 0.004 ^ab^	0.598 ± 0.010 ^b^	759.31 ± 45.82 ^bcde^	0.250 ± 0.008 ^a^
20/40 kHz	76.56 ± 0.21 ^a^	2.35 ± 0.07 ^a^	1.42 ± 0.25 ^a^	0.73 ± 0.25 ^a^	80.23 ± 1.63 ^b^	1253.77 ± 51.11 ^b^	0.757 ± 0.140 ^b^	0.581 ± 0.007 ^b^	551.43 ± 104.73 ^e^	0.234 ± 0.014 ^a^
25/28 kHz	76.46 ± 0.36 ^a^	2.37 ± 0.07 ^a^	1.43 ± 0.23 ^a^	0.65 ± 0.34 ^a^	73.77 ± 1.44 ^e^	1743.38 ± 28.52 ^a^	0.903 ± 0.004 ^ab^	0.597 ± 0.013 ^b^	939.84 ± 25.94 ^b^	0.255 ± 0.010 ^a^
25/40 kHz	77.06 ± 1.13 ^a^	2.29 ± 0.12 ^a^	1.80 ± 0.52 ^a^	1.35 ± 1.20 ^a^	83.90 ± 2.12 ^ab^	1157.94 ± 93.26 ^b^	0.906 ± 0.030 ^a^	0.618 ± 0.040 ^b^	648.34 ± 70.46 ^de^	0.268 ± 0.030 ^a^
28/40 kHz	77.85 ± 1.08 ^a^	2.16 ± 0.10 ^a^	2.07 ± 0.50 ^a^	2.17 ± 1.19 ^a^	75.83 ± 0.83 ^de^	1613.44 ± 47.45 ^a^	0.905 ± 0.013 ^ab^	0.600 ± 0.039 ^b^	876.10 ± 63.79 ^bc^	0.261 ± 0.022 ^a^
28/68 kHz	77.32 ± 1.35 ^a^	2.01 ± 0.09 ^b^	1.77 ± 0.62 ^a^	1.68 ± 1.33 ^a^	80.77 ± 1.02 ^bc^	1727.91 ± 173.74 ^a^	0.887 ± 0.010 ^ab^	0.569 ± 0.011 ^b^	872.08 ± 89.85 ^bcd^	0.230 ± 0.011 ^a^
40/68 kHz	76.45 ± 0.34 ^a^	2.16 ± 0.08 ^a^	1.49 ± 0.19 ^a^	0.71 ± 0.32 ^a^	83.03 ± 1.32 ^ab^	1828.40 ± 47.03 ^a^	0.901 ± 0.004 ^ab^	0.589 ± 0.031 ^b^	970.31 ± 57.02 ^b^	0.249 ± 0.013 ^a^

Values are presented as mean ± standard deviation (*n* = 3). Different lowercase letters within the same column indicate significant differences among treatments (*p* < 0.05). “-” indicates that the value was not applicable because Δ*E* was calculated relative to the control group.

**Table 2:** Particle size distribution of beef myofibrillar protein emulsions under dual-frequency ultrasound-assisted glycation.

Treatment	Size Distribution (nm)	
	Small-Particle Peak	Large-Particle Peak
Control	636–1318	\
20/25 kHz	204–391	2324–19,095
20/28 kHz	636–811	\
20/40 kHz	498–1121	\
25/28 kHz	174–260	1681–7224
25/40 kHz	498–811	\
28/40 kHz	240–391	8495–19,095
28/68 kHz	174–811	\
40/68 kHz	260–540	1550–19,095

“\” indicates that no distinct large-particle peak was detected.

## Data Availability

The data presented in this study are available from the corresponding authors upon reasonable request.
